# Supplementary material for: A fault-intrusion-tolerant system and deadline-aware algorithm for scheduling scientific workflow in the cloud
Source: PeerJ Comput Sci. 2021 Nov 2;7:e747. doi: 10.7717/peerj-cs.747 (PMC8576554; doi:10.7717/peerj-cs.747)
Supplement: Supplemental Information 6 [file peerj-cs-07-747-s006.zip › Myproject1.1/docs/org/workflowsim/ClusteringEngine.html]

ClusteringEngine


JavaScript is disabled on your browser.


- Overview
- Package
- Class
- Use
- Tree
- Deprecated
- Index
- Help

- Prev Class
- Next Class

- Frames
- No Frames

- All Classes

- Summary:
- Nested |
- Field |
- Constr |
- Method

- Detail:
- Field |
- Constr |
- Method


org.workflowsim

## Class ClusteringEngine

- java.lang.Object
- - org.cloudbus.cloudsim.core.SimEntity
  - - org.workflowsim.ClusteringEngine

- All Implemented Interfaces:
  :   java.lang.Cloneable

  ---

    

  ```
  public final class ClusteringEngine
  extends SimEntity
  ```

  ClusteringEngine is an optional component of WorkflowSim and it merges tasks
  into jobs

  Since:
  :   WorkflowSim Toolkit 1.0

- - ### Field Summary

    Fields

    | Modifier and Type | Field and Description |
    | --- | --- |
    | `protected int` | `cloudletsSubmitted` The number of tasks submitted. |
    | `protected BasicClustering` | `engine` The clustering engine to use |
    | `protected java.util.List<Job>` | `jobList` The job list |
    | `protected java.util.List<Task>` | `taskList` The task list |
    | `protected java.util.List<? extends Task>` | `taskReceivedList` The task received list. |
    | `protected java.util.List<? extends Task>` | `taskSubmittedList` The task submitted list. |

    - ### Fields inherited from class org.cloudbus.cloudsim.core.SimEntity

      `FINISHED, HOLDING, RUNNABLE, WAITING`
  - ### Constructor Summary

    Constructors

    Constructor and Description

    `ClusteringEngine(java.lang.String name,
    int schedulers)`

    Created a new ClusteringEngine object.
  - ### Method Summary

    Methods

    | Modifier and Type | Method and Description |
    | --- | --- |
    | `protected void` | `finishExecution()` Send an internal event communicating the end of the simulation. |
    | `java.util.List<Job>` | `getJobList()` Gets the job list. |
    | `java.util.List<Task>` | `getTaskList()` Gets the task list. |
    | `java.util.List<Task>` | `getTaskReceivedList()` Gets the task received list. |
    | `java.util.List<Task>` | `getTaskSubmittedList()` Gets the tasks submitted list. |
    | `WorkflowEngine` | `getWorkflowEngine()` returns the WorkflowEngine |
    | `int` | `getWorkflowEngineId()` returns the WorkflowEngineId |
    | `protected void` | `processClustering()` Processes events available for this ClusteringEngine. |
    | `protected void` | `processDatastaging()` Adds data stage-in jobs to the job list |
    | `void` | `processEvent(SimEvent ev)` Processes events available for this Broker. |
    | `protected void` | `processOtherEvent(SimEvent ev)` Overrides this method when making a new and different type of Broker. |
    | `protected void` | `setJobList(java.util.List<Job> jobList)` Sets the job list. |
    | `protected void` | `setTaskList(java.util.List<Task> taskList)` Sets the task list. |
    | `protected void` | `setTaskReceivedList(java.util.List<Task> taskReceivedList)` Sets the task received list. |
    | `protected void` | `setTaskSubmittedList(java.util.List<Task> taskSubmittedList)` Sets the tasks submitted list. |
    | `void` | `shutdownEntity()` This method is invoked by the `Simulation` before the simulation finishes. |
    | `void` | `startEntity()` This method is invoked by the `Simulation` class when the simulation is started. |
    | `void` | `submitTaskList(java.util.List<Task> list)` This method is used to send to the broker the list of cloudlets. |

    - ### Methods inherited from class org.cloudbus.cloudsim.core.SimEntity

      `cancelEvent, clone, getEventBuffer, getId, getName, getNextEvent, getNextEvent, getState, numEventsWaiting, numEventsWaiting, pause, run, schedule, schedule, schedule, schedule, scheduleFirst, scheduleFirst, scheduleFirst, scheduleFirst, scheduleFirstNow, scheduleFirstNow, scheduleFirstNow, scheduleFirstNow, scheduleNow, scheduleNow, scheduleNow, scheduleNow, selectEvent, send, send, send, send, sendNow, sendNow, sendNow, sendNow, setEventBuffer, setId, setState, waitForEvent`
    - ### Methods inherited from class java.lang.Object

      `equals, finalize, getClass, hashCode, notify, notifyAll, toString, wait, wait, wait`

- - ### Field Detail


    - #### taskList

      ```
      protected java.util.List<Task> taskList
      ```

      The task list


    - #### jobList

      ```
      protected java.util.List<Job> jobList
      ```

      The job list


    - #### taskSubmittedList

      ```
      protected java.util.List<? extends Task> taskSubmittedList
      ```

      The task submitted list.


    - #### taskReceivedList

      ```
      protected java.util.List<? extends Task> taskReceivedList
      ```

      The task received list.


    - #### cloudletsSubmitted

      ```
      protected int cloudletsSubmitted
      ```

      The number of tasks submitted.


    - #### engine

      ```
      protected BasicClustering engine
      ```

      The clustering engine to use
  - ### Constructor Detail


    - #### ClusteringEngine

      ```
      public ClusteringEngine(java.lang.String name,
                      int schedulers)
                       throws java.lang.Exception
      ```

      Created a new ClusteringEngine object.

      Parameters:
      :   `name` - name to be associated with this entity (as required by
          Sim\_entity class from simjava package)
      :   `schedulers` - the number of schedulers in this ClusteringEngine

      Throws:
      :   `java.lang.Exception` - the exception
  - ### Method Detail


    - #### getWorkflowEngineId

      ```
      public int getWorkflowEngineId()
      ```

      returns the WorkflowEngineId


    - #### getWorkflowEngine

      ```
      public WorkflowEngine getWorkflowEngine()
      ```

      returns the WorkflowEngine


    - #### submitTaskList

      ```
      public void submitTaskList(java.util.List<Task> list)
      ```

      This method is used to send to the broker the list of cloudlets.

      Parameters:
      :   `list` - the list


    - #### processClustering

      ```
      protected void processClustering()
      ```

      Processes events available for this ClusteringEngine.

      Parameters:
      :   `ev` - a SimEvent object


    - #### processDatastaging

      ```
      protected void processDatastaging()
      ```

      Adds data stage-in jobs to the job list

      Parameters:
      :   `$none` -


    - #### processEvent

      ```
      public void processEvent(SimEvent ev)
      ```

      Processes events available for this Broker.

      **Specified by:**
      :   `processEvent` in class `SimEntity`

      Parameters:
      :   `ev` - a SimEvent object


    - #### processOtherEvent

      ```
      protected void processOtherEvent(SimEvent ev)
      ```

      Overrides this method when making a new and different type of Broker.
      This method is called by `#body()` for incoming unknown tags.

      Parameters:
      :   `ev` - a SimEvent object


    - #### finishExecution

      ```
      protected void finishExecution()
      ```

      Send an internal event communicating the end of the simulation.


    - #### shutdownEntity

      ```
      public void shutdownEntity()
      ```

      **Description copied from class: `SimEntity`**

      This method is invoked by the `Simulation` before the simulation finishes. If you want
      to save data in log files this is the method in which the corresponding code would be placed.

      **Specified by:**
      :   `shutdownEntity` in class `SimEntity`


    - #### startEntity

      ```
      public void startEntity()
      ```

      **Description copied from class: `SimEntity`**

      This method is invoked by the `Simulation` class when the simulation is started. This
      method should be responsible for starting the entity up.

      **Specified by:**
      :   `startEntity` in class `SimEntity`


    - #### getTaskList

      ```
      public java.util.List<Task> getTaskList()
      ```

      Gets the task list.

      Type Parameters:
      :   `T` - the generic type

      Returns:
      :   the task list


    - #### getJobList

      ```
      public java.util.List<Job> getJobList()
      ```

      Gets the job list.

      Type Parameters:
      :   `T` - the generic type

      Returns:
      :   the job list


    - #### setTaskList

      ```
      protected void setTaskList(java.util.List<Task> taskList)
      ```

      Sets the task list.

      Type Parameters:
      :   `T` - the generic type

      Parameters:
      :   `taskList` - the new task list


    - #### setJobList

      ```
      protected void setJobList(java.util.List<Job> jobList)
      ```

      Sets the job list.

      Type Parameters:
      :   `T` - the generic type

      Parameters:
      :   `jobList` - the new job list


    - #### getTaskSubmittedList

      ```
      public java.util.List<Task> getTaskSubmittedList()
      ```

      Gets the tasks submitted list.

      Type Parameters:
      :   `T` - the generic type

      Returns:
      :   the task submitted list


    - #### setTaskSubmittedList

      ```
      protected void setTaskSubmittedList(java.util.List<Task> taskSubmittedList)
      ```

      Sets the tasks submitted list.

      Type Parameters:
      :   `T` - the generic type

      Parameters:
      :   `taskSubmittedList` - the new task submitted list


    - #### getTaskReceivedList

      ```
      public java.util.List<Task> getTaskReceivedList()
      ```

      Gets the task received list.

      Type Parameters:
      :   `T` - the generic type

      Returns:
      :   the task received list


    - #### setTaskReceivedList

      ```
      protected void setTaskReceivedList(java.util.List<Task> taskReceivedList)
      ```

      Sets the task received list.

      Type Parameters:
      :   `T` - the generic type

      Parameters:
      :   `taskReceivedList` - the new cloudlet received list


- Overview
- Package
- Class
- Use
- Tree
- Deprecated
- Index
- Help

- Prev Class
- Next Class

- Frames
- No Frames

- All Classes

- Summary:
- Nested |
- Field |
- Constr |
- Method

- Detail:
- Field |
- Constr |
- Method
